# Acknowledgement to Reviewers of *Biosensors* in 2014

**DOI:** 10.3390/bios5010025

**Published:** 2015-01-07

**Authors:** Biosensors Editorial Office

**Affiliations:** MDPI AG, Klybeckstrasse 64, CH-4057 Basel, Switzerland

The editors of *Biosensors* would like to express their sincere gratitude to the following reviewers for assessing manuscripts in 2014:

A. Lewis, Robert
Althoefer, Kaspar
Balland, Martial
Basu, Partha
Bedioui, Fethi
Berret, Jean-françois
Bian, Chao
Biswal, Sibani Lisa
Bormio Nunes, Cristina
Brutschy, Malte
Bubb, Daniel-dennis M.
Burda, Hynek
Chang, Sheng-Po
Chen, Jiping
Chen, Min
Chen, Tzong-Yueh
Chichkov, Boris
Chou, Tse-Chuan
Chrisey, Douglas B.
Claeyssens, Frederik
Convertine, Anthony
Curley, G. Paul
De-Juan-Pardo, Elena M.
Delahaut, Philippe
Dhumpa, Raghuram
Du, Sarah
Duda, Katarzyna
Emilio, Morallon
Ertl, Peter
Fernandes, Elisabete
Fry, Albert J.
Fujita, Takayuki
Gao, Zhiqiang
Glass, Timothy E.
Golden, Glen
Goltsev, Vasilij
Goto, Hiromasa
Greer, James A.
Guo, Ting
Guo, Ya
Halámek, Jan
Harms, Hauke
Harren, Frans J. M.
Holland, Richard
Holmes-Smith, A. Sheila
Hore, Peter J.
Hou, Yanglong
Huang, Long-sun
Huguet, Silvia Soria
Inagi, Shinsuke
Jeons, Sangmin
Ju, Heongkyu
Jung, Friedrich
Jungner, Högne
Kang, Seong Ho
Kang, Shin-Won
Karande, Pankaj
Kennedy, Ian
Killard, Tony
Kim, Jungkyu
Kim, SeungHyun
Knopp, Dietmar
Kuehn, E. Wolfgang
Kumar, Challa V.
Lagarde, Florence
Landers, James
Lee, Jong Eun
Lee, Kwan Hyi
Lee, Youngkwan
Leipziger, Jens
Leucht, Philipp
Leung, Chung-Hang
Lin, Hung-Yin
Liu, Qingjun
Long, Rong
Lou, Xia
Ma, Edmond Dik-Lung
Maeda, Isamu
Majidi, Carmel
Mamotte, Cyril D.
Mannik, Jaan
Maróti, Péter
Mcewan, Alistair
Mihara, Hisaaki
Miki, Hirofumi
Miosge, Nicolai
Moscone, Danila
Muratore, Christopher
Neuman, Keir
Nöll, Gilbert
Onodera, Takeshi
Othéro de Brito, Vera Lúcia
Oukarroum, Abdallah
Pablo Salvador, Juan
Park, Hun-kuk
Payne, Gregory F.
Pedrero, Maria
Phillips, John
Phuoc, Nguyen N.
Piro, Benoit
Posthuma, Truus
Pyun, Jae-chul
Qi, Zhimei
Reed, Scott M.
Rodriguez-Hernandez, Juan
Russell, Alan J.
Saunders, Rachel E.
Secco, Emanuele
Selleri, Stefano
Selvakumar, Sagaya
Shen, Wen
Skladal, Petr
Stine, O. Colin
Striegler, Susanne
Tamura, Toshiyo
Tkac, Jan
Tombelli, Sara
Tovar, Günter
Turner, Nicholas
Vashist, Sandeep K.
Vass, Imre
Velotta, Raffaele
Venditti, Iole
Vilcinskas, Andreas
Wagner, Patrick
Waldvogel, Siegfried R.
Wang, Junbo
Wedge, Christopher
Weinert, Emily
Weißbecker, Bernhard
Wróblewski, Wojciech
Wu, Peter Ka-Chai
Xing, Da
Yan, Steve
Yang, Catherine F.
Yang, Shikuan
Yoon, Young Jin
Zeng, Hulie
Zhang, Haijun
Zhang, Qichun
Zhou, Xiao Dong
Zhu, Xiaoshan

